# Applying Network Theory to Epidemics: Control Measures for *Mycoplasma pneumoniae* Outbreaks

**DOI:** 10.3201/eid0902.020188

**Published:** 2003-02

**Authors:** Lauren Ancel Meyers, M.E.J. Newman, Michael Martin, Stephanie Schrag

**Affiliations:** * Santa Fe Institute, Santa Fe, New Mexico, USA;; † University of Texas at Austin, Austin, Texas, USA;; ‡ University of Michigan, Ann Arbor, Michigan, USA;; § Centers for Disease Control and Prevention, Atlanta, Georgia, USA

**Keywords:** epidemiology, models, theoretical, network, respiratory tract infections, *Mycoplasma pneumonia*

## Abstract

We introduce a novel mathematical approach to investigating the spread and control of communicable infections in closed communities. *Mycoplasma pneumoniae* is a major cause of bacterial pneumonia in the United States. Outbreaks of illness attributable to mycoplasma commonly occur in closed or semi-closed communities. These outbreaks are difficult to contain because of delays in outbreak detection, the long incubation period of the bacterium, and an incomplete understanding of the effectiveness of infection control strategies. Our model explicitly captures the patterns of interactions among patients and caregivers in an institution with multiple wards. Analysis of this contact network predicts that, despite the relatively low prevalence of mycoplasma pneumonia found among caregivers, the patterns of caregiver activity and the extent to which they are protected against infection may be fundamental to the control and prevention of mycoplasma outbreaks. In particular, the most effective interventions are those that reduce the diversity of interactions between caregivers and patients.

Mathematical modeling has a rich and growing tradition in epidemiology (1-3). Because experimental approaches to epidemic interventions are often impractical, and in some cases unethical, mathematical models can provide otherwise unobtainable insights on the spread and control of disease. Recently, considerable interest has been shown in the effect of contact networks on the spread of disease, and particularly in using the so-called percolation theory to model epidemics (4-10). Agent-based simulation is also being used increasingly to help epidemiologic investigations (11). In this paper, we use both of these tools to assess the effects of epidemic interventions in closed health-care facilities.

*Mycoplasma pneumoniae* is a major cause of bacterial pneumonia in the United States (12). This bacterium, the smallest self-replicating organism capable of cell-free existence, is spread both by direct contact between an infected person and a susceptible person, and by airborne droplets expelled when an infected person sneezes, coughs, or talks. Large, sustained outbreaks of *M. pneumoniae* have occurred in closed and semi-closed populations such as hospitals, psychiatric institutions, military and religious communities, and prisons (13-15). Public health officials and health-care providers struggle, often with little success, to control mycoplasma outbreaks because of the long incubation period of the organism, late detection of outbreaks, and an incomplete understanding of the effectiveness of various infection control strategies.

Effective measures to control mycoplasma outbreaks are needed to limit the associated illness and substantial costs. Previous work has addressed candidate strategies, including infection control practices to prevent the exchange of respiratory droplets between patients and caregivers, cohorting members of the community who display symptoms of a respiratory infection, and antibiotic prophylaxis of asymptomatic members of the community (14-16). The costs of these strategies include curtailed social interactions because of cohorting, undesirable side effects or allergic reactions to prophylactic antibiotics, and a potential increase in the risk for infections caused by antibiotic-resistant bacteria. Studies of these control measures have been limited by incomplete information and participation.

Using a network model approach, we show how data on interactions in real-world communities can be translated into graphs—mathematical representations of networks—and how to predict the course of an epidemic from the structure of a graph. We found that the assignment of caregivers to patient groups is more critical to the course of an epidemic than the cohorting of patients. Within our models, the most effective interventions are those that reduce the diversity of interactions that caregivers have with patients. For example, an institution with many wards can avoid a large outbreak by confining caregivers to work in only one or very few wards.

## The Model

**Figure 1:**
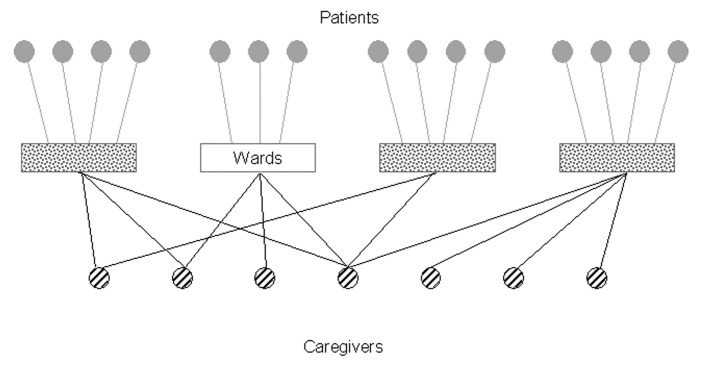
Health-care institution network. Each vertex represents a patient, caregiver, or ward, and edges between person and place vertices indicate that a patient resides in a ward or a caregiver works in a ward.

Here we model an institution with spatially disjointed wards. Patients are confined to a single ward, and caregivers work in one or more wards. Each person or ward is represented by a “vertex” in the graph. “Edges” connect people to the wards in which they reside or work. Figure 1 shows the graph for an institution with four wards, each with three or four patients and two to four caregivers.

A key property of graphs is their degree distribution. The degree of a vertex is the number of other vertices to which it is connected. In Figure 1, for example, the degree of all patients is one; the degree of each caregiver ranges from one to four; and the degree of the wards ranges from six to seven, indicating the number of inhabitants and caregivers working there. Direct transmission of *M. pneumoniae* can only occur between two vertices if an edge connects them.

Throughout this model, we allow transmission to occur between people and places. We do not mean that bacteria actually infect a space by residing on inanimate objects or in the air. Rather, we mean that the person has transmitted the bacteria to another person who resides or works in that place. Conversely, when a place transmits to a person, we mean that the bacterium is transmitted to an uninfected person living or working in that place.

We begin by considering only the caregivers and wards. Later we add the patients to the model. (All notations are defined in the Table.) A probability generating function (pgf) is a mathematical quantity that describes a probability distribution, and thereby summarizes a large amount of useful information about the network architecture. We can define pgfs that capture the distribution of the number of wards assigned to each caregiver and the distribution of the number of caregivers working in each ward.

**Table:** Notation for epidemiologic interaction network model

Notation	Definition
*W*	Number of wards in the facility
*C*	Number of caregivers working in the facility
*μ_w_*	Average no. of caregivers working in a ward
*μ_c_*	Average no. of wards in which a caregiver works
*r*	Probability that a given caregiver works in a given ward
*Ρ_χ_*	Probability that a caregiver works in *k* wards
*q_χ_*	Probability that a ward has *k* caregivers working in it
ƒ_0_(*x*)	Probability generating function (pgf) for the degree distribution of caregivers
g_0_(*x*)	pgf for the degree distribution of wards
ƒ_1_(*x*)	First select a random ward, and then select a random caregiver working there. This expression represents the pgf for the number of other wards in which that caregiver works.
g_1_(*x*)	First select a random caregiver, and then select a random ward associated with that caregiver. This expression represents the pgf for the number of other caregivers working in that ward.
*τ_w_*	Probability of transmission from a ward to a caregiver
*τ_c_*	Probability of transmission from a caregiver to a ward
Ф_0_(x)	pgf for the number of wards affected by transmission from a random caregiver
Ф_1_(x)	First select a random ward and assume that it is affected by the bacterium, then select a random caregiver working there. This expression represents the pgf for the number of other wards affected by that caregiver.
Γ_0_(*x*)	pgf for the number of caregivers affected by transmission from a random ward
Γ_1_(*x*)	First select a random caregiver and assume he/she is infected, then select a random ward in which that caregiver works. This expression represents the pgf for the number of other caregivers infected by individuals working/living in that ward.
_ 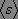 _	Average number of wards affected in an outbreak
1 - *Ѕ_c_*	The size of the caregiver giant component—the largest set of infected caregivers that are all connected through work in common wards
*2- Ѕ_w_*	The size of the ward giant component—the largest set of affected wards that are all connected through common caregivers
β_w_(*x*)	pgf for the number of patients in affected ward *w* who contract the bacterium
*Β*(*x*)	pgf for the total number of patients in the facility who are infected during an epidemic

Pgfs can be mathematically manipulated to give many useful results. For example, the derivative gives the average of the distribution, e.g., the mean number of wards assigned to a caregiver, or the mean number of caregivers working in a ward. We can also answer the following question using pgfs: If an infected caregiver exposes a ward, how many other caregivers, on average, will be vulnerable to infection because they also work in that ward? Appendix A defines our pgfs and describes the derivations that answer this question.

### Transmission through the Graph

Transmission of *M. pneumoniae* occurs when people occupy the same physical space for some period of time. Therefore, in our model, transmission can occur between persons if the vertices representing them are connected to the same ward.

We derive two complementary estimates for the size of an outbreak. The first is appropriate for conditions not conducive to large outbreaks, such as a pathogen with low transmissibility, or an institution with few interpersonal interactions. The second applies to conditions that favor large outbreaks.

We begin with two questions. If a healthy caregiver works in an infected ward, how many other wards will eventually become infected as a result of that caregiver’s interaction with that ward? Similarly, if an infected caregiver works in a yet uninfected ward, how many other caregivers will eventually become infected as a result of that caregiver’s activity in that ward? Answers to these questions vary from ward to ward and from caregiver to caregiver. Therefore, we calculate probability distributions for the spread, which we represent by using pgfs.

**Figure 2:**
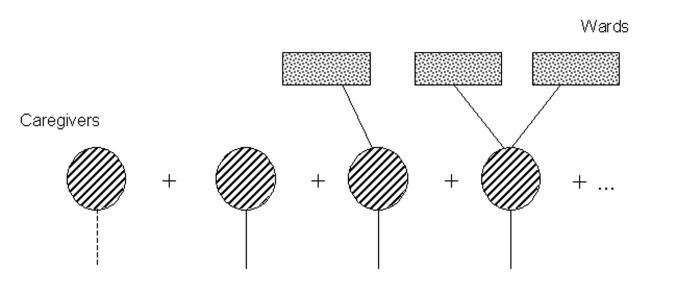
Future transmission diagram I, summing all possible future transmissions stemming from a caregiver who works in an infected ward.

First, consider an edge linking an infected ward to a caregiver. Figure 2 breaks down the possible scenarios. First, the caregiver may not become infected. Second, the caregiver might become infected but not transmit to any other wards. Third, the caregiver might transmit infection to one or more other wards in which he or she works. In Appendix B, we construct a pgf by summing up the probabilities of these different outcomes.

**Figure 3:**
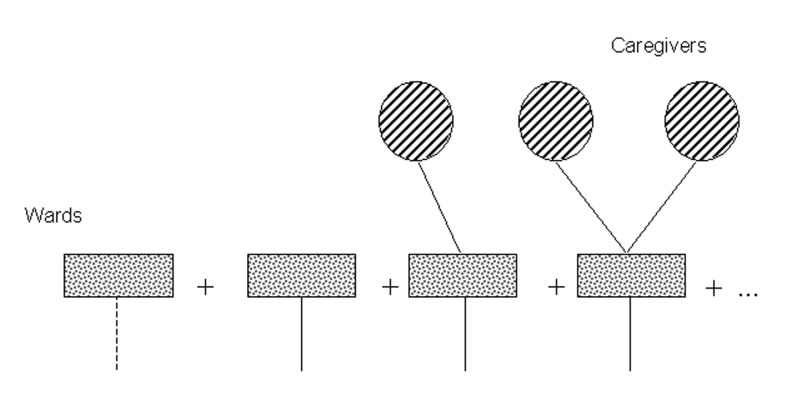
Future transmission diagram II, summing all possible future transmissions stemming from a ward in which an infected caregiver works.

Next, we start with an edge from an infected caregiver to a ward. As shown in Figure 3, there may be no transmission along the edge in question to the ward, no further transmission from the ward to other people, or transmission to one or more other people who spend time in the ward.

With these two pgfs, we derive the average size of a small outbreak, starting from a single infection:

_
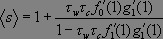
_
[1]

Where ƒ′ denotes the first derivative of *ƒ* with respect to its argument. Thus, the average size of the outbreak is 1 (the original patient) plus a function of the two transmission rates (from caregivers to wards, *τ_c_* , and from wards to caregivers, *τ_w_*), and the average number of wards assigned to a caregiver ( ƒ′_0_(1) ). The term ƒ′_1_(1) assumes that we choose any ward at random from the entire network, then choose one of the edges connected to that ward at random, then follow that edge to a caregiver, and finally calculate the number of other wards assigned to the caregiver. On average, that will be ƒ′_1_(1). Likewise g′_1_(1) is the average number of other caregivers working in a ward that we reach by first choosing a caregiver at random and then randomly choosing one of the wards in which the caregiver works. These terms contain information not only about the average degrees of caregivers and wards but also about the probability that a given caregiver or node will become infected in the first place.

The expression for _
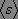
_ diverges when

*τ_w_τ_c_ƒ′_1_*(1)*g′_1_*(1) = 1 [2]

This expression represents the transition between a regime in which only small isolated outbreaks of disease can occur and one in which a full-blown community-wide epidemic can occur. A community will cross that transition point if transmission rates are sufficiently high (*τ_w_* and *τ_c_*) or the interactions among wards and caregivers are sufficiently dense (_ƒ_′_1_(1)and g′_1_(1) ). Equation no. 1 provides an estimate of the epidemic size below the threshold only. It is based on the assumption that interactions are rare enough that a person or a place only encounters the infection once. When interactions are more common and the community lies above the epidemic transition, we must use a different estimate for the size of the outbreak.

The “giant component” of the graph is the largest connected set of vertices that have all been infected. The size of the outbreak above the epidemic transition is exactly equal to the number of vertices in this giant component. We calculate the size of the giant component *Ѕ_c_* (the number of caregivers affected) by calculating the fraction of vertices not contained in it:

1 - *Ѕ_c =_* Ф_0_(1) , [3]

where Ф_0_(1) is the probability that an infected caregiver will produce no further infections (Appendix B). A similar expression describes the number of wards affected in an epidemic:

1-*Ѕ*_w_= Γ _0_ (1). [4]

These expressions reflect both the fraction of the population infected and the probability that an outbreak will reach epidemic proportions in the first place. Since *Ѕ_c_* and *Ѕ_w_* are often much less than 1, not all outbreaks turn into epidemics, even above the epidemic transition.

### Degree Distributions

Equation nos. 3 and 4 allow us to estimate the size of an epidemic on the basis of transmission probabilities and the degree distribution of caregivers to wards. To make specific numerical predictions, we must first calculate pgfs for the degree distributions. Here we make the simple assumption that the degree distributions follow a Poisson distribution for both the number of wards associated with a given caregiver and the number of caregivers associated with a given ward. This assumption is equivalent to requiring that all caregivers have an equal likelihood of working in any ward and that a caregiver is assigned to any given ward independent of his or her other ward assignments. In the absence of more specific information about assignment to wards, this assumption seems a reasonable first step. This distribution assumes an infinite population and is generally applied to very large populations. Although perhaps not the ideal model for small institutions, this distribution is used here because it yields pgfs with convenient mathematical properties (see Appendix C).

## Case Study

Data gathered by the Centers for Disease Control and Prevention (CDC) during a recent mycoplasma outbreak allowed us to extract values for the parameters in our theory. In 1999, an outbreak of mycoplasma pneumonia occurred in a psychiatric institution (14). All 15 wards at the institution were affected, with 60 of 257 residents and 82 of 440 employees diagnosed with mycoplasma-like illness. In the following sections, we predict the epidemic threshold for this institution. The threshold is a function of the degree distribution of caregivers and transmission rates, the size of the epidemic above the threshold, and a range of realistic transmission rates for *M. pneumoniae* in this outbreak.

We assumed that each patient was confined to a single ward. While this was not true for all patients at the institution, it simplified the mathematics and allowed us to make a reasonable approximation of the epidemiology. Interactions between patients in separate wards will increase the threat of a full-blown epidemic and make early intervention all the more critical. Including such interactions in the model is possible by adding edges to the graph that connect patients to multiple wards. This scenario can be solved exactly by using techniques similar to those presented here.

### Epidemic Threshold

If we assume that the degree distributions for wards and caregivers are Poissonian, the epidemic threshold (equation no. 2) is equivalent to τ_w_ τ_c_ μ_w_μ_c_=1.

In other words, when the product of the transmission rates, the average number of caregivers per ward, and the average number of wards per caregiver exceeds 1, epidemics become possible. In the psychiatric institution, *W* = 15 and *C* = 440, hence _
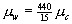
_ and the threshold becomes _
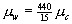
_.

**Figure 4:**
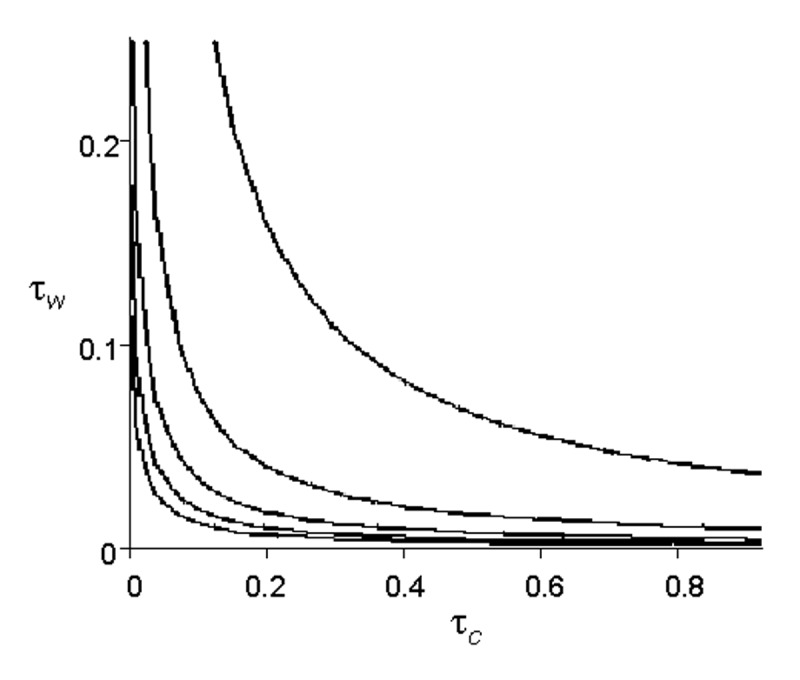
Epidemic thresholds. Each line assumes a different value for *μ_c_*(the average number of wards per caregiver), and graphs the combination of *τ_c_* and *τ_w_*(transmission parameters) above which the population crosses the epidemic threshold. From top to bottom, the lines represent *μ_c_*= 1, *μ_c_*= 2, *μ_c_*= 3, *μ_c_*= 4, and *μ_c_*= 5 .

Figure 4 illustrates the epidemic threshold for five different demographic scenarios ( μ_c_ = 1,2,3,4,5 ). For the most densely connected case, when each caregiver works in five wards on average, the epidemic threshold is crossed at very low rates of transmission. When the community is less densely connected, it can withstand much higher infectivity without giving rise to epidemics.

### Calculating the Size of the Epidemic

Combining equation no. 2 with equations 5, 6, 7, and 8 from Appendix C, we derived the following:

Ф_0_(1) = exp[*μ_w_* (1- *τ_c +_ τ_c_* exp[ *μ_c_*( 1-*τ_w +_ τ_w_*Ф_0_(1) - 1 ] -1) ]. [9]

Given values for demographic parameters *μ_c_* and *μ_w_* , we search for the value of Ф_0_(1) that satisfies equation no. 9 numerically. Then, the predicted number of caregivers infected during an epidemic is *Ѕ_c_ = 1-* Ф_0_(1). (The number of affected wards is similarly derived.) Since we know neither the exact distribution of caregivers in wards nor the transmission rates between caregivers and wards, we solve for the size of the epidemic outbreak in a range of values of the three independent parameters *μ_c_*, *τ_c_* and *τ_w_* .

**Figure 5:**
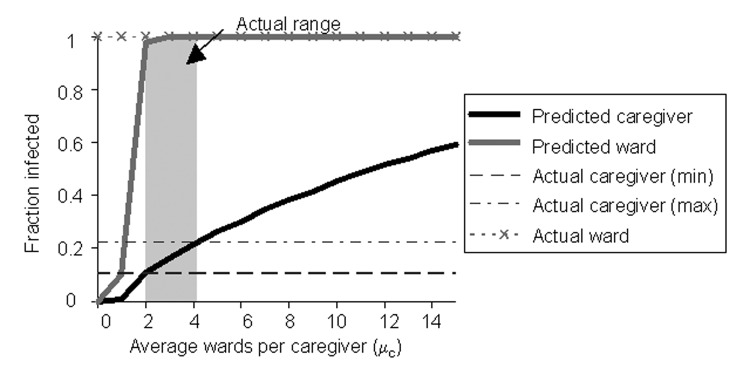
Size of epidemic. Predicted and actual number of caregivers and wards affected in an outbreak. These predictions assume that the transmission rate from caregivers to wards is *τ_c_* = 0.6 and from wards to caregivers is *τ_w_*= 0.06.

Figure 5 shows both the fraction of wards and caregivers infected in our model as a function of the number of wards per caregiver ( *μ_c_*), and the fraction of wards and caregivers infected in the actual outbreak. We assume transmission rates of *τ_c_ = 0.6* and *τ_w_ =0.06* (discussed below). The top dashed line indicates that 100% of the wards were affected during the actual epidemic. The lower horizontal lines depict the upper and lower bound empirical estimates for the number of caregivers affected (TB Hyde, unpub. data). As *μ_c_* increases, so does the possibility of transmission from one ward to another through caregivers that work in both. The number of wards affected climbs sharply to 100% (as actually occurred in this outbreak), whereas the number of caregivers climbs more gradually, passing through the realistic range at relatively low values of *μ_c_*.

This analysis suggests that the likelihood of an epidemic and the eventual size of an epidemic, should one occur, are highly sensitive to the degree distribution for caregivers. Transmission of *M. pneumoniae* is limited, and the extent and duration of the outbreak are reduced if each caregiver’s activities are confined to just a few wards.

The derivations given here are exact in the limit of large network size. To assess their accuracy on networks like these with a few hundred vertices, we have constructed specific graphs that realize these distributions and performed computer simulations of the spread of epidemics on them. Each simulation constructs a network with 15 wards and 440 caregivers, where the degree distribution of each caregiver is binomial with *n* = 15 , and p such that *n*p = *μ_c_*. We assume constant infection periods of *δ*_c_= 14 days (for caregivers) and *δ*_w_ = 21 days (for wards) and that contact between a caregiver and a ward occurs independently of any other such contact. Initially a single, randomly chosen caregiver is infected. Every day, transmission occurs from an infected caregiver to a connected ward with probability *τ_c_*. Thus, the probability that the caregiver will transmit the infection to the connected ward at all is _
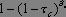
_. Likewise, the daily transmission rate from an affected ward to a healthy caregiver that works there is _
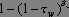
_.

**Figure 6:**
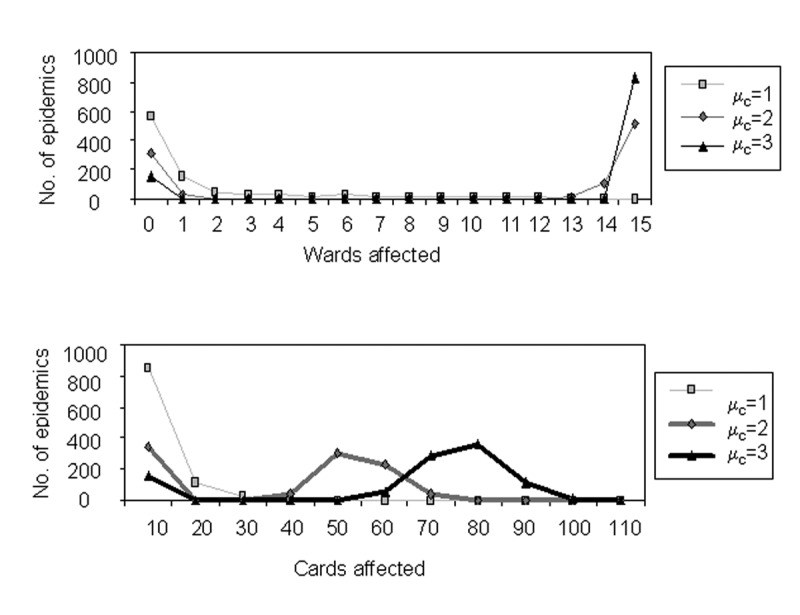
Simulated outbreak sizes. Frequency distributions of the numbers of wards and caregivers affected in 1,000 epidemic simulations are shown for *μ_c_*= 1,2,3.

**Figure 7:**
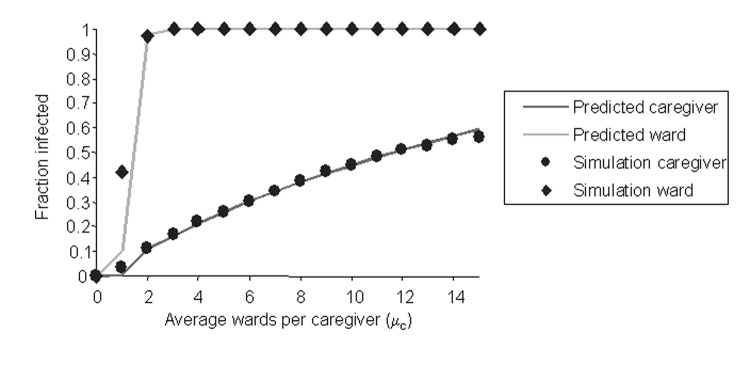
Comparing derivations to simulation. This graph compares the analytical predictions to the size of a simulated outbreak averaged over 1,000 simulations for each value of *μ_c_*.

Figure 6 shows a frequency distribution of the sizes of epidemics for 1,000 runs of the simulation. Figure 7 compares these results with the predictions of our analytic theory. As the figure clearly shows, the agreement between simulation and theory is excellent.

### Inferring Transmission Rates

Our numeric method also allows us to pinpoint transmission rates that are consistent with the empirical observations. Assuming the average caregiver works in one to four wards, we identify transmission rates that predict the observed numbers of affected caregivers and wards. We find that *τ_c_* ∈ [0.2,1] and *τ_w_*∈[0.03,0.1]. Transmission from an infected caregiver to at least one patient in a ward must therefore be about 10 times more likely than transmission from a ward with sick patients to a caregiver who works in that ward. Remarkably, caregivers are not likely to become infected, yet when they are infected, they become the primary vehicles for spreading bacteria from ward to ward. Hence the most effective interventions will be those that prevent transmission to caregivers.

### The Patients

**Figure 8:**
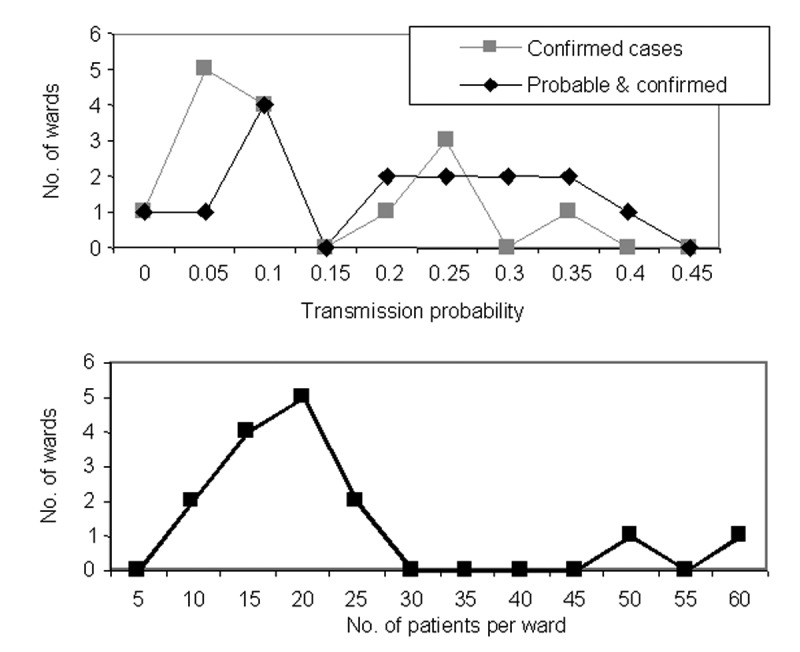
Distribution of transmission rates and ward sizes in the psychiatric institution.

Based on the outbreak data, the probability that a particular patient will become infected if at least one other patient in the ward is infected is 0.15 (0.02) for confirmed cases or 0.23 (0.02) when probable cases are included.^1^ Figure 8 shows the within-ward transmission rates and ward size for the 15 wards. Although not shown, ward size and the transmission rate are not correlated.

**Figure 9:**
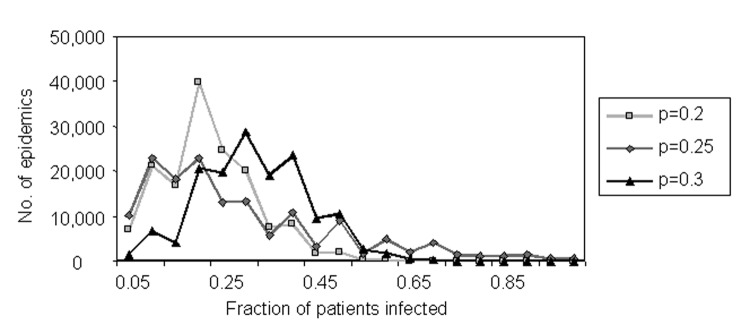
Simulated spread of *Mycobacterium pneumoniae* among patients within a ward.

We simulate the spread of *M. pneumoniae* among patients, assuming the ward size distribution shown in Figure 8, and assuming that the number of patients infected per ward follows a binomial distribution with probability parameter p. (The Poisson approximation is inappropriate as it only applies to very large wards with small transmission rates.) That is, all 15 wards are assumed to be affected, and each patient in a ward becomes infected with probability p. Figure 9 shows frequency distributions for the fraction of patients infected in 100,000 simulations at three values of p (p = 0.2,0.25,0.3). These distributions resemble the actual frequency distribution shown in Figure 8, and thereby support the binomial approximation.

## Discussion

Network theory enables epidemiologists to model explicitly and analyze patterns of human interactions that are potential routes for transmission of an infectious disease. The statistical properties of an epidemic graph determine the extent to which an infectious agent can spread. By manipulating the structure of a graph, we can identify interventions that may dramatically alter the course of an epidemic, or even prevent one altogether, and translate them into measures that make sense in a real community. In this paper, we have used network methods to model the spread of a respiratory tract infection in a health-care facility.

How might this be applied to a real outbreak? We have considered data from a recent investigation of an outbreak of *M. pneumoniae* in a residential psychiatric institution (14). In that investigation, standard infection control practices, including strict respiratory droplet precautions, cohorting of ill patients, and employee education about mycoplasma illness and symptoms were instituted at the facility. Unfortunately, *M. pneumoniae* has a long incubation period (1–4 weeks), during which time an asymptomatic, infected person can transmit the bacterium to an uninfected person. This long incubation period limits the beneficial effect of cohorting, since infected persons are only identified and taken out of the community after they have passed through the incubation period.

In both the outbreak and our model (assuming parameters based on this particular institution), caregivers are less likely to become infected than are patients. This observation may mislead investigators and lead to inappropriate recommendations. Although caregivers are less likely to become ill, they are the primary vectors of infection in the facility. Our model suggests that transmission rates from patients to caregivers are lower than transmission rates from caregivers to patients. Therefore, once a caregiver is infected with *M. pneumoniae*, the likelihood is high that they will transmit the infection to their patients. These data support infection control strategies that limit transmission of *M. pneumoniae* to caregivers.

We suggest two complementary strategies: limit the number of wards with which caregivers interact, and reduce the probability that caregivers become infected through, for example, respiratory droplet precautions. This strategy limits the time and cost of laboratory testing as well as the risks for antibiotic use in uninfected persons. The activity of some ancillary staff (e.g., physical therapists and nutritionists) cannot be limited to a select number of wards. In these cases, alternative precautions against transmission of *M. pneumoniae* are required.

We conclude with three caveats. First, the epidemic model includes all infections, even those that do not result in symptoms. Most persons with *M. pneumoniae* infections have relatively mild disease, only a cough or sore throat or no symptoms at all (17). When applying the model to the outbreak investigation, we considered only symptomatic carriers. While including asymptomatic carriers would change the estimates for the rates of transmission, our qualitative recommendations for intervention would remain the same.

Second, for mathematical tractability, our model assumes random (Poissonian) assignment of caregivers to wards. The quantitative (but probably not qualitative) results would differ under different degree distributions. In the future, we hope to analyze distributions taken from actual health-care institutions, when available.

Third, because of the long incubation period of *M. pneumoniae* infection, interventions are often initiated well into the outbreak. Since epidemics can last months, and in the psychiatric institution at least half of the wards were not affected until 6 weeks after the first case-patient was diagnosed, we are optimistic that intervention of the type proposed will have a positive impact.

The theoretical tools are in place for building community-specific networks and analyzing the transmission of infectious diseases on these networks. Our approach enables mathematical experiments, in which the inputs are interventions—structural reorganization, cohorting, treatment, and the like—and the output is predictions about the spread of a disease (or lack thereof) on the network. This approach can both aid the development of general measures and lend insight into specific scenarios in which intervention is still possible.

## Appendix A

### Probability Generating Functions

Let *Ρ_χ_* be the normalized probability that a randomly chosen caregiver is working in *k* wards and *q_χ_* the probability that a randomly chosen ward has *k* caregivers working in it. We define probability generating functions (pgfs) for these degree distributions thus:

Caregivers: ƒ_0_(*x*) = ∑*Ρ_Χ_Χ^Χ^*

Wards: *g*_0_(Χ) = ∑*q_χ_Χ^Χ^* .

Since *Ρ_χ_* and *q_χ_* are each properly normalized probability distributions, _
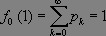
_ and *g*_0_(1)=1. The generating functions contain all the same information as the probability distributions but in a form that will be more convenient for our purposes. We can always recover the probability distributions again by differentiation _
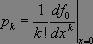
_.

If we assume that each of *W* wards has on average *μ_w_* caregivers working in it, and each of *C* caregivers interact with *μ_c_* wards on average, then, ƒ′_0_(1) = *μ_c_* and g′_0_(1) = *μ_w_*. (In general, the moments of the probability distributions are given by derivatives of the generating functions evaluated at one.)

Suppose we now choose a caregiver at random and follow an edge to a ward in which the caregiver works. The pgf for the number of caregivers working this ward is _
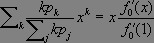
_. Hence the distribution of caregivers working in this ward *other* than the originally selected caregiver is described by _
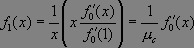
_.

Likewise, if we start from a specific ward and choose a random caregiver working in that ward, then the number of *other* wards in which the caregiver works is given by _
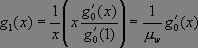
_.

## Appendix B

### Deriving _
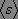
_ and *Ѕ_c_*

We denote the probability of transmission from a caregiver to a ward as *τ_c_* and the probability of transmission from a ward to a caregiver as *τ_w_*. By summing the probabilities for the different outcomes depicted in Figure 2, we arrive at a generating function for the number of wards that will ultimately be affected:

Ф_1_(*x*) = (1-*τ_w_*) + *τ_w_x*p̃_1_ + *τ_w_x*p̃_2_Γ_1_(*x*) + … = (1-*τ_w_*) + *τ_w_x*ƒ_1_ (Γ_1_ (*x*)),

where p̃_i_ is the probability that the caregiver transmits the infection to *i* new wards. Each term in this expression corresponds to a pictorial term in Figure 2. Recall that ƒ_1_(*x*) is a generating function for the number of wards with which a caregiver interacts (other than the ward from which transmission occurred). Γ_1_(*x*) is the generating function (discussed below) for the number of future infections starting with an edge going from a caregiver to a chosen ward. The generating function for the number of infections starting with a randomly chosen infected caregiver is Ф_0_(*x*) = *x*ƒ_0(_Γ_1_(*x*)).

Next, the generating function for the cluster of infections arising from a randomly chosen edge from a person to a ward is thus Γ_1_(*x*) = (1-*τ_c_*) + *τ_c_*(*g*_1_ (Ф_1_(*x*))) and Γ_0_(*x*) = *g*_0_ (Ф_1_(*x*)).

Substituting into the formulas for Ф_0_(x) and Ф_1_(x), we find Ф_0_(x) = *Χ*ƒ_0_[1-*τ_c_* + *τ_c_*g*_1_*(Ф_1_(*x*))] and Ф_1_(*x*) = 1 -*τ_w_* + *τ_w_x*ƒ_1_ [1- *τ_c_*+ *τ_c_g_1_*(Ф_1_(*x*))]. To calculate average outbreak size _
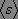
_, we differentiate Ф_0_(1):

_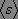 =_ Ф′_0_(1)= ƒ_0_(1-*τ_c_* + *τ_c_g_1_*(1)) +ƒ′_0_ (1- *τ_c_* + *τ_c_g_1_*+ (1))*τ_c_*g′_1_(1)Ф′_1_(1) = 1 + *τ_c_*ƒ′_0_(1) g′_1_(1)Ф′_1_(1)

Now, solving for Ф′_1_(x), we find Ф′_1_(x) = *τ_w_*ƒ_1_[ 1- *τ_c+_ τ_c_*g_1_(Ф_1_(*x*))] + *τ_w_x*ƒ′_1_[ 1 - *τ_c_*+ *τ_c_*g_1_(Ф_1_(*x*))]∙*τ_c_*g′_1_ (Ф_1_(*x*))∙ Ф′_1_(*x*). Hence, _
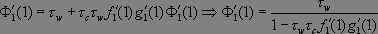
_. We thereby arrive at the following expression for average outbreak size:

_
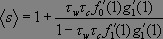
_.

Turning next to the size of the giant component, we know that 1 - *Ѕ_c =_* Ф_0_(1) = ƒ_0_(1 -*τ_c_* + *τ_c_*g_1_(Ф_1_(1). Hence *Ѕ_c_* = 1 – ƒ_0_(1 -*τ_c_* + *τ_c_*g_1_(Ф_1_(1). Likewise 1 - *Ѕ_w_* = Γ_0_(1) = g_0_(1-*τ_w_* +*τ_w_ƒ_1_* ( Γ_1_(1)) implies *Ѕ_w_* = 1 – g_0_(1 - *τ_w_*+*τ_w_ƒ_1_* (Γ_1_ (1)).

## Appendix C

### The Poisson Generating Function

If the probability that a given caregiver works in some ward is *r*, then the generating function for the number of wards per caregiver would be

_
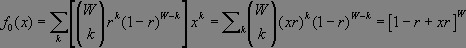
_.

Substituting for *r* , we find _
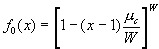
_. In the limit of a large number of wards, the binomial distribution approaches a Poisson distribution, and the generating function for the Poisson distribution is

_
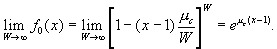
_  [5]

Likewise, in the limit of many caregivers, g_0_ (*x*) =*e^μw^* (x-1). [6]

Performing a bit more mathematical legwork, we find that


_
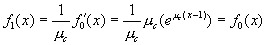
_
[7]


and similarly g_1_(*x*) = g_0_(*x*). Note also that if we know the values of *W*,*C* , and *μ_c_*, we can derive the average number of caregivers per ward:

_
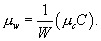
_
[8]

## Footnotes

1 We calculate these rates by averaging the fraction of infected patients per ward across the 15 wards and compute the error by taking the standard deviation of these fractions, divided by the square root of the sample size.
